# Reversing the trend of weak policy implementation in the Kenyan health sector? – a study of budget allocation and spending of health resources versus set priorities

**DOI:** 10.1186/1478-4505-5-3

**Published:** 2007-03-29

**Authors:** Anna H Glenngård, Thomas M Maina

**Affiliations:** 1 The Swedish Institute for Health Economics (IHE), P.O. Box 2127, 220 02 Lund, Sweden; 2 Institute for Policy Analysis and Research (IPAR), P.O. Box 45843-00100, GPO, Nairobi, Kenya

## Abstract

**Background:**

Policy implementation in the context of health systems is generally difficult and the Kenyan health sector situation is not an exception. In 2005, a new health sector strategic plan that outlines the vision and the policy direction of the health sector was launched and during the same year the health sector was allocated a substantial budget increment. On basis of these indications of a willingness to improve the health care system among policy makers, the objective of this study was to assess whether there was a change in policy implementation during 2005 in Kenya.

**Methodology:**

Budget allocations and actual expenditures compared to set policy objectives in the Kenyan health sector was studied. Three data sources were used: budget estimates, interviews with key stakeholders in the health sector and government and donor documentation.

**Results:**

Budget allocations and actual expenditures in part go against policy objectives. Failures to use a significant proportion of available funds, reallocation of funds between line items and weak procurements systems at the local level and delays in disbursement of funds at the central level create gaps between policy objectives and policy implementation. Some of the discrepancy seems to be due to a mismatch between responsibilities and capabilities at different levels of the system.

**Conclusion:**

We found no evidence that the trend of weak policy implementation in the Kenyan health sector was reversed during 2005 but ongoing efforts towards hastening release of funds to the districts might help solving the issue of low absorption capacity at the district level. It is important, however, to work with clear definitions of roles and responsibilities and well-functioning communications between different levels of the system.

## Background

Health systems are subject of competing and conflicting goals and information asymmetry between different actors, resulting in resistance to systems change [1]. Hence, implementing policies or priorities in the context of health systems is difficult. In the context of Sub-Saharan Africa, public health care has gone through a period of increasing shortage of resources for health since the early 1980s. Important problems of public health systems in these countries are services of poor quality, inequitable distribution of resources and services, inadequate procurement systems and inefficient supervision coupled with a high disease burden [2-4].

Kenya does not seem to be an exception when it comes to difficulties of implementing policies in the health care sector, although policy documents are well supported by accurate data [5-8]. Implementation of the first National Health Sector Strategic Plan 1999–2004 (NHSSP I) was far from accomplished. The NHSSP I emphasised the need to prioritise primary heath care. The allocation of health resources, however, is skewed in favour of the tertiary and secondary facilities that offer curative services. Still, rural health facilities are usually the first point of contact with patients and provide the major bulk of the health care services [7,9]. Another example is the failure to meet the set target of allocating 15 percent of total government spending to the health sector as agreed in the Abuja declaration. Possible explanations of the weak policy implementation during the last decade include insufficient human and financial resources, unrealistic policy targets and that the health sector has suffered from a lack of political commitment, operational management of strategies and means to evaluate implementation of policies as well as poor communication between different levels of the system [5,8,10,11].

Policy implementation can be studied from the perspective of budgetary processes [12]. Policy makers should ideally use budgeting as a tool to help implement policies by understanding the current financial position and setting the best course for the future. Studying budget decisions enables researchers to make concrete comparisons between budget allocations and policy objectives. "Perhaps the 'study of budgeting' is just another expression for the 'study of politics' [...] The opportunities for comparison are ample, the outcomes are specific and quantifiable" (Wildavsky 1974, p 3).

What makes a budgetary system is the interaction between spending and cutting roles according to Wildavsky, 1986 [13]. Roles or the expected behaviour in relation to institutional position is part of the division of labour. "Administrative agencies act as advocates of increased expenditure, and central control organs function as guardians of the treasury" (Wildavsky 1986, p 11–12). This definition of roles is similar to that in agency theory, commonly used to analyse actors within health care systems (see e.g. Dranove & White 1987; Gauri 2001; Gauri et al 2004 [4,14,15]). In the context of health care systems, providers can be seen as agents or advocates and the funding body as principal or guardian.

The behaviour of the actors in budgetary systems in different countries is affected by overall wealth and predictability in the budget environment. Wildavsky, 1986 describe budgetary processes in different countries based on these two dimensions. Wealth refers to differences in the gross national product and predictability refers to the degree of uncertainty a country is faced with in terms of resources available for spending versus demand for spending. Budgetary poverty implies an inability to generate adequate resources and budgetary uncertainty implies an inability to predict the flow of expenditures and/or revenues in the near future [13].

## Objective

The objective of this paper is to assess policy implementation from the perspective of budget allocations and actual expenditures in the context of the health care sector in a poor country. The study is limited to the case of the health care system of Kenya, more specifically whether there was a change in the Kenyan governments allocation and spending of health care resources in relation to their set priorities in distribution of funds in 2005/06 compared to previous years.

Whether the situation of weak policy implementation in the Kenyan health sector was reversed during 2005 is particularly interesting to study since the second Kenyan National Health Sector Strategic Plan (NHSSP II) 2005–2010 was launched in 2005. The new plan is said to be practically operational and was based on consensus among policy makers to a higher degree than the NHSSP I was [11]. Moreover, for the financial year 2005/06 (the Kenyan Financial Year runs from July to June) the Kenyan health sector was approved a budget increase of 30 percent. These two facts indicate a willingness to improve the health care system in the country among policy makers.

## Kenya – the case

Kenya is a low-income country situated in Sub-Saharan Africa. There are 8 provinces and 71 districts in the country and together with the MoH headquarter they form the basis of the health care system. The major health care provider is the MoH, which operates more than half of all health facilities in the country. The public delivery system is organised in a traditional pyramidal structure. First level care is provided at dispensaries and medical clinics. The next level is the health centres and sub-district hospitals. Third level care is provided at district hospitals and provincial general hospitals. There are two national hospitals; Moi Referral and Teaching Hospital in Eldoret and Kenyatta National Hospital, located in Nairobi [16].

Resources for health are scarce and the disease burden is high in the country, just as in other countries in the region. The estimated total per capita expenditure on health was USD 19.2 in 20001/02, which is about half of what is required to finance the minimum health package set by the World Health Organization [13]. The shortage of resources for health applies also to real resources. There are about 5,000 doctors in Kenya, for a population of 32 million, i.e. about 6,400 inhabitants per doctor [11]. The major source of funding is the households (51%), followed by the government (30%) and donors (16%) [16].

## Methods

Yin (2003) suggests using multiple sources of data in a case study for the purpose of triangulation of evidence [17]. The results in this study are based on both quantitative and qualitative data. Multiple sources (archival records interviews and documentation) of data were used.

Budget figures (archival records) were used to provide concrete examples of budget allocations and actual expenditures on health based on hard data. According to Yin (2003) the advantage of using such data is that they are precise and quantitative and that they exist prior to the study [17]. Preliminary and approved budgets and actual expenditure data was collected from the Ministry of Finance (MoF) and Ministry of Health (MoH). Preliminary or printed budgets are estimates that are to be released by the MoF to all different sectors in June for the upcoming year. Approved or revised budgets constitute the actual amounts that are to be disbursed during the ongoing year and are to be released by the MoF in February.

In total, 12 interviews where conducted with key stakeholders in the Kenyan health sector. As noted by Yin (2003) interviews are one of the most important sources of information in a case study [17]. The respondents were selected based on their knowledge about and involvement in the in the Kenyan health sector. All respondents had senior positions within their organisations and are regarded to be key informants [17] representing the Kenyan health sector. Due to the position they occupied within their respective organisation and their extensive knowledge and experience in the Kenya's health sector, the information generated was assumed to be representative of the views of the groups, which the interviewees represented and was therefore regarded sufficient to provide a holistic picture of the problem being investigated. They represented the MoF (three respondents), the MoH (three respondents), multilateral organisations (two respondents) and bilateral donor organisations (four respondents). The interviews were conducted in Nairobi, Kenya, April 19–27, 2006.

The interviews were semi-structured and based on open-ended questions of both substantive and theoretical nature [18]. The purpose of the substantive questions was to capture the specific features of the Kenyan resource allocation system and the purpose of the theoretical questions was to link the features of policy implementation in the context of the Kenyan health care system to findings in previous studies.

A review of government and donor documentation, e.g. National Health Accounts and Public Expenditure Reviews, was also undertaken to support the findings from the interviews. The documents were collected during the interviews and through searches at the website of the government of Kenya.

## Results

### The budgetary process in Kenya

The budget process in Kenya is made within the framework of the Medium Term Expenditure Format (A MTEF is a tool for linking policy, planning and budgeting over a medium-term at the government level. It consists of a top-down resource envelope and a bottom-up estimation of the current and medium term costs of existing policies [19]). The MoF sets ceilings for all sectors on a three-year basis. The different ministries participate in working groups where sector priorities are reviewed and harmonised with national priorities. The MoH present a health sector review, which should reflect needs of hospitals and districts, upon which their bidding process is based. In reality, however, incremental budgeting on a top-down principle is practised. Figures are not based on costed activity plans for service delivery but on available resources from the central level, i.e. the MoF. Funds allocated to the MoH are in turn allocated to the districts and hospitals by the MoH headquarters with no clear linkages between the districts and the MoH headquarter [20].

The MoF releases printed (preliminary) budgetary estimates for the upcoming year to all sectors in June. Based on these estimates, the MoH prepares an allocation plan that outlines the distribution of expected funds further down to the districts. In February, the MoF should release the revised (approved) budgets. There is one recurrent component and one development component in the budget for all sectors. The recurrent component should cover running costs at health centres, dispensaries and hospitals.

The MoH control the health budget and release funds to the districts and the two national hospitals. The allocation to the districts for health centres and dispensaries is in the form of line-item budgets whereas the hospitals receive global budgets. The development component is to be released on a biannual basis and the recurrent component on a quarterly basis. Salaries to staff are paid for directly by the MoH. Drugs are also procured centrally, by the Kenya Medical Suppliers Agency (KEMSA) and then delivered to the districts [21].

The mode for allocating resources is through Authorities to Incur Expenditures (AIEs) – a government payment guarantee. The AIEs are issued by the MoH and then paid for by the MoF through the district treasury. There is no possibility for the districts or the MoH to spend funds that were allocated for one financial year during another period. Funds that are not spent by the end of June are returned to the MoF [22].

Not all funding for the health sector is channelled through the MoH. Many donors choose to channel funds outside the MoH controlled system in order to avoid bottlenecks in terms of slow disbursement of funds at the central level. Donors also have control and reporting requirements parallel to those by the MoH [20].

### Kenyan health policy objectives and targets

The second Kenyan National Health Sector Strategic Plan 2005–2010 (NHSSP II) was launched in 2005 with the theme "Reversing the Declining Health Trends". The NHSSP II defines the vision of the Kenyan health care system as *"Achievement of an efficient and high quality health care system that is accessible, equitable and affordable for every Kenyan household" *and the mission as *"To promote and participate in the provision of integrated and high quality promotive, preventive, curative and rehabilitative health care services to all Kenyans"*. The strategy is to strengthen primary health care services in order to facilitate the provision of low-cost and accessible services in rural areas [11].

In terms of resource allocation, according to the NHSSP II, resources should be focused at; a) preventive and promotive health services; b) rural dispensaries and health centres; c) expansion of primary health care; and d) implementing appropriate policy, financial and organisational reforms. Among other things, resources should be shifted from curative towards preventive services [11]. Related to financial and organisational reforms, the MoH together with collaborating partners are in the process of preparing for a Sector Wide Approach (SWAp) for health in Kenya.

In the Public Expenditure Review (PER) 2005 specific targets for reallocation of expenditures in order to focus priority programmes are set at [21]:

• Increasing expenditures on rural health facilities and preventive health by 15 percent;

• Increasing allocation for drugs for rural health facilities by 18 percent; and

• Reducing expenditures on Kenyatta National Hospital by 3.8 percent.

### Budget allocation

The 30 percent increase in the printed budget corresponds to Kshs 6,542 million (approximately USD 96 million). According to interviews with senior staff at the MoH, the increased budget was earmarked for primarily five areas:

• Kshs 800 million was earmarked for drugs at all three levels of care

• Kshs 720 million was earmarked for equipment at all three levels of care. All dispensaries and health centres should first receive a minimum set of equipment to be able to deliver such services that are required by first level care facilities. Then, hospitals should receive whatever is left according to their priorities.

• Kshs 1 000 million was earmarked for rehabilitation of facilities. The mode for allocating these funds was similar as that for equipment, i.e. hospitals are getting whatever is left once health centres and dispensaries have received their part.

• Kshs 290 million was earmarked for immunisation.

• Kshs 211 million was earmarked for reproductive health.

**Figure 1:**
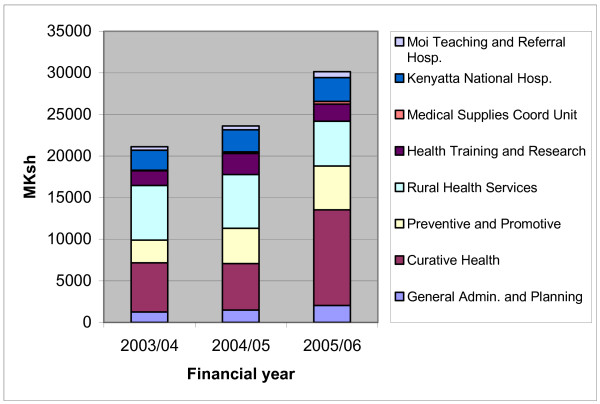
Allocation of printed health budget 2003/04-2005/06, million KSh. Source: MTEF 2006/07-2008/09 [19].

Disaggregated figures covering the year 2005/06 are only available for the printed budgets. The revised budgets that should have been available in February were still not decided in April when the collection of data for this study took place. Actual expenditures will not be available until the financial year is over. Thus, comparison over time based on hard data is only possible using the preliminary budget allocation for 2005/06. In Figure 1 the allocation of printed budgets for different items is illustrated for the financial years 2003/04, 2004/05 and 2005/06. The data is presented in absolute numbers.

Both in terms of proportion of budget and in terms of actual funds, curative care account for the largest increase in allocation when comparing the printed figures over time. This shows a mismatch between policy pronouncements, where primary care is prioritised according to the MoH but is allocated minimal resources (see Figure 1). The fact that allocation of resources for health is skewed in favour of tertiary and secondary care is also observed in published reports [7,9].

### Budget implementation

There are weaknesses in budget implementation when printed estimates are compared with revised estimates and actual expenditures. Generally, there are considerable gaps between the printed and approved budgets and actual expenditures, and specifically for the development component. There is an inability to spend available funds at both central and local levels. This applies to both government and other funding [22,23]. Often, districts tend to spend funds from those donors who pose the least conditions in terms of controlling and reporting, according to the interviews. Moreover, in a financial disbursement assessment report from 2004 it is concluded that there is a failure to use a significant proportion of available donor resources in the health sector [23].

For the financial year 2005/06, data on actual expenditure is available up to quarter two and only in terms of development and recurrent components. Comparing the first two quarters 2005/06 with the same period the previous year indicates an increased ability to spend. Actual expenditures as a proportion of allocated resources increased from below 80 percent to slightly above 100 percent over the period (see Table 1).

**Table 1:** Actual expenditures as percentage of target second quarter 2004/05 and 2005/06

	**Q2 2004/05**	**Q2 2005/06**
Recurrent	96.0%	100.9%
Development	6.4%	88.7%
Total	77.9%	100.5%

Source: Quarterly budget reviews, Second Quarter 2004/2005 and 2005/2006 [21–22]

In order to cope with funding gaps at district level, during the financial years 2003/04 and 2004/05 funds were reallocated from rural health and preventive and promotive services when comparing approved budgets with actual expenditures. The allocation to curative health constituted no more than 28 and 24 percent respectively in the printed budgets whereas it constituted 49 and 46 percent respectively of actual expenditures (see Table 2).

**Table 2:** Allocation to different line items – printed and approved budget and expenditures 2003/04-2004/05 and printed budget 2005/06, million KSh (percentage of health budget)

	**2003/04**			**2004/05**			**2005/06**
	**Printed**	**Approved**	**Expenditures**	**Printed**	**Approved**	**Expenditures**	**Printed**

General Admin. and Planning	1 262 (6.0)	1 157 (5.8)	957 (5.8)	1 494 (6.3)	1 536 (7.0)	1 382 (7.2)	2 047 (6.8)
Curative Health	5 911 (28.0)	5 445 (27.4)	7 975 (48.5)	5 590 (23.7)	9 122 (41.5)	8 802 (45.9)	11 497 (38.1)
Preventive and Promotive	2 724 (12.9)	2 038 (10.2)	952 (5.8)	4 241 (18.0)	3 217 (14.6)	1 730 (9.0)	5 261 (17.4)
Rural Health Services	6 571 (31.1)	6 720 (33.8)	2 134 (13.0)	6 443 (27.3)	3 086 (14.1)	2 508 (13.1)	5 380 (17.8)
Health Training and Research	1 770 (8.4)	1 602 (8.1)	1 525 (9.3)	2 558 (10.8)	1 751 (8.0)	1 488 (7.8)	2 074 (6.9)
Medical Supplies Coord Unit	70 (0.3)	69 (0.3)	32 (0.2)	169 (0.7)	135 (0.6)	133 (0.7)	322 (1.1)
Kenyatta National Hospital	2 398 (11.4)	2 409 (12.1)	2 409 (14.7)	2 659 (11.3)	2 659 (12.1)	2 659 (13.9)	2 858 (9.5)
Moi Teaching and Referral Hospital	422 (2.0)	458 (2.3)	458 (2.8)	458 (1.9)	458 (2.1)	458 (2.4)	714 (2.4)
Total MoH	21 127 (100.0)	19 898 (100.0)	16 442 (100.0)	23 611 (100.0)	21 964 (100.0)	19 158 (100.0)	30 153 (100.0)

Source: MTEF 2006/07-2008/09 [19]

Although the printed proportion for rural health services for the financial year 2005/06 is small compared to the two previous years, it is higher than the actual expenditure compared to the same period. This implies that if the budget for 2005/06 is translated into actual expenditures, there will be a positive development regarding reallocations of funds from curative care towards preventive and promotive care (see Table 3).

**Table 3:** Comparison of allocation to different line items – printed budget and expenditures 2004/05 and printed budget 2005/06, million KSh (percentage of health budget)

	**Change 2005/06 to 2004/05**	
	**Printed 05/06 vs. printed 04/05**	**Printed 05/06 vs. expenditures 04/05**

General Adm. and Planning	37.0%	48.1%
Curative Health	105.7%	30.6%
Preventive and Promotive	24.1%	204.1%
Rural Health Services	-16.5%	114.5%
Health Training and Research	-18.9%	39.4%
Medical Supplies Coord Unit	91.1%	142.8%
Kenyatta National Hospital	7.5%	7.5%
Moi Teaching and Referral Hospital	55.9%	55.9%

Total MoH	27.7%	57.4%

Source: MTEF 2006/07-2008/09 [19]

When comparing the printed figures for the financial years 2005/06 and 2004/05 there is no evidence that the increment in the latest budget is actually used according to priorities. Comparing actual expenditures in 2004/05 with the printed budget for 2005/06, however, indicates a change according to stated priorities. Even though the *printed *proportion for rural health services is small in 2005/06 compared to the two previous years, it is higher than the *actual expenditure *compared to the two previous years (see Table 4).

**Table 4:** Policy targets and actual change 2004/05 to 2005/06

	**Change 2004/05 to 2005/06**		**Target**
	**Printed 05/06 vs. printed 04/05**	**Printed 05/06 vs. expenditures 04/05**	

Preventive and Promotive	-0.5%	8.4%	15.0%
Rural Health Services	-9.4%	4.8%	15.0%
Kenyatta National Hospital	-1.8%	-4.4%	-3.8%

Source: MTEF 2006/07-2008/09 [19]

#### Identified problems with the ability to spend

Problems related to the inability to spend available funds listed in the financial disbursement assessment report from 2004 include [23]:

- A lack of information about development resources available at district level;

- The MoH only release a fraction of the approved development budget;

- Fragmented financial planning and allocation processes;

- A lack of qualified staff at the department of finance, MoH;

- Poor financial and management reporting systems and;

- Procurement difficulties at all levels.

Donors experiment with alternative allocation mechanisms to avoid bottlenecks at MoH and MoF and donor conditionality for project support does not converge among different donors. Furthermore, the decentralisation process that started a decade ago has so far not been successful in the case of Kenya. The efforts towards implementing a decentralised system have led lead to a poor match between responsibilities and capabilities at different levels of the public health system [23]. According to the interviews, local authorities are weak in terms of managing capabilities and control over financial resources. The definition of roles and responsibilities has not been clear.

Interviews with the key informants indicated that the gap between allocated funds and actual expenditures is largely due to a slow release of funds and poor procurement system, which causes delays in spending. Regarding slow release of funds, the planning capacity is weak at all levels of the system, according to the interviews with MoH staff. At the ministry level, the process of disbursement of funds is slow, which causes uncertainty for the providers and impede their planning process. Slow release of funds to the districts has been a bottleneck to expenditures at the district level. To tackle this issue of low absorption capacity, the MoF allowed for funds to be disbursed on pre-financing arrangements in 2005, whereby facilities receive an AIE accompanied with a cheque and this is expected to facilitate implementation of programmes and increase actual expenditures [21].

Slow procurement is a planning problem at all levels, but particularly at the district level, according to respondents at ministry level. Due to weak confidence regarding the size and time of the actual release of funds, district level managers await funding before they procure services instead of procuring services and pay as funding arrives. Since the release of funds usually is delayed, procurement is delayed and in the end there are positive balances on accounts at the end of the financial year. The current system does not allow for the MoH or the districts to spend funds that were allocated for one specific year in another period. Funds that are not spent by June 30^th ^are automatically returned to the MoF. At the district level there is not only weak confidence in the financing system, including the flow of funds, but also in suppliers of drugs and materials. The delivery of drugs and materials is viewed as supply-driven in the sense that the districts do not always receive what they request but rather what KEMSA have in store [22].

The authors of the financial disbursement assessment report from 2004 concluded that there are serious problems in the financial disbursement system, resulting in a failure to use a significant proportion of available financial resources and that these problems cannot be solved by minor changes. The problems are deeply rooted in the system and management culture [23]. Other stakeholders in the health sector share this view, according to the interviews with key informants.

## Discussion

Public health care in Sub-Saharan Africa has gone through a long period of increasing shortage of human as well as financial resources for health. Inadequate procurement systems, public service delivery that is not accountable and inefficient supervision are important problems of public systems in poor countries [2,3]. According to the results in this study, Kenya is no exception.

Increased resources were provided for the financial year 2005/06, which gave policy makers an opportunity to allocate increased funds towards prioritised areas without necessarily cutting from other areas. The allocation of funds from the central level in the Kenyan health care sector, however, goes in part against set policies and the reallocations of funds between line items at district level creates further deviations between set policies and implementation. Also in a previous study of health sector reforms in Kenya it was concluded that there is a large gap between policy formulation and implementation [8]. In the current study it was found that that there are considerable gaps between the printed and approved health budgets and actual health expenditures in general and for the development component in specific. A large part of the gap is explained by delays in the allocation of funds and a slow procurement system, resulting in an inability to spend allocated funds. Funds that are not spent by the end of the financial year cannot be carried forward and spent in another period. Moreover, a reallocation of funds towards curative services has taken place. This reallocation has meant that allocated funds are spent against policy directions and prioritised areas since it is clearly articulated that resources should be allocated towards primary and promotive services. Thus, an increased ability to spend allocated funds might lead to a higher degree of policy implementation. Efforts towards a quicker release of funds through disbursements on pre-financing arrangements, which was introduced in 2005, might improve the absorption capacity at the district level.

Scarce resources for health make it in practice difficult for policy makers to prioritise one area without cutting from other areas. In a resource constrained environment, reallocating funds inevitably creates 'winners and losers', and thereby tensions [26,27]. But even allocating extra funds can create tensions as those who do not receive as large increments as others perceive themselves as losers. Implementing unpopular decisions requires both capabilities and incentives among those with the task of implementation. Failure of implementing reallocations can often be expressed in terms of failure of will as decision makers lack the will of implementing unpopular decisions [28].

Franco et al, 2002, define incentives as the "will-do" component and capabilities as the "can-do" component and of individual motivation [29]. Capabilities relate to the extent to which resources including skills are mobilised to enable the achievement of set goals or priorities. Incentives relate to the extent to which human resources adapt to those goals and are affected primarily through feedback related to job performance and work culture. Strengthened strategic and operational capacities are necessary for the capability to act. But having the capability to act is not similar to having the incentives to act. Both capabilities and incentives are necessary conditions for change [29-31].

Current budgeting practises constitute a problem of gaps and delays in allocation and disbursement of funds at the district level. Thereby the control over financial resources at district level becomes weak. Further, weak managing and planning capabilities and a poor procurement system carry an inability to actually spend disbursed funds, i.e. the "can-do" component seems to be weak. The problems might be related to an unclear definition of roles and responsibilities at as well as poor communication between different levels of the system. The current top-down approach to the planning and budgeting system means that funds allocated to districts and hospitals by the central level with no clear linkages between the districts and the MoH. Thereby the districts are given directions that do not necessarily coincide with their preferences or perceived priorities, i.e. the incentives of implementing budgets set by the central level or the "will-do" component might be weak. Also in a previous study of Kenya it was found that, in order to achieve desired outcomes of health policy processes, systematic management, monitoring and evaluation of processes are crucial [8].

The interaction between spending and cutting roles constitutes a budgetary system [13]. In poor countries, the definition of roles might be even more important than in rich countries.

According to Wildavsky (1986), in poor and uncertain countries, repetitive budgeting is found. Finance ministries in these countries generally approve budgetary estimates ex ante but when it is time to disburse the funds payments are delayed. Poverty carries a delay in the disbursement of money and uncertainty carries a need to reprogram funds repeatedly to adjust to the changing conditions in the system [13]. In such countries, it is often easier to work with improved roles and responsibilities rather than overall budgetary poverty and uncertainty. As Gauri (2001) suggest, in developing countries with poor quality and inequitable distribution of health care services, a key function of the funding body is to define responsibilities of different types of providers and levels of care, and organise the available resources accordingly [4].

## Conclusion

Policy implementation in the context of the Kenyan health sector has been weak for a long period of time. The first National Health Sector Strategic Plan (1999–2004) was not successfully implemented and the governments proportion of expenditure in relation to total government spending is far below the agreed target in the Abuja declaration. In this paper the aim was to explore whether year 2005 was the year of reversing the trend of weak policy implementation. Even if the will to change is there among policy makers as a new strategic plan for the health sector was launched and the health sector was approved a substantial budget increment this year, this study found no evidence of reversing the trend. Budget allocations from the central level partly goes against policy objectives and a reallocation of funds at the district level creates even wider gaps between policy objectives and actual spending. Some of the discrepancy can be explained by a mismatch between responsibilities and capabilities at different levels of the system, particularly in terms of low absorption capacity at district level. Ongoing efforts towards achieving a quicker release of funds to lower levels of the system are one way of tackling this issue. What is important, however, is to work with clear definitions of roles and responsibilities and well-functioning communications between different levels of the system.

## Authors' contributions

AG participated in the collection and analysis of data and writing of the manuscript. TM participated in the collection and analysis of data, and assisted in the writing of the manuscript.
